# Where Have the Periods Gone? The Evaluation and Management of Functional Hypothalamic Amenorrhea

**DOI:** 10.4274/jcrpe.galenos.2019.2019.S0178

**Published:** 2020-02-06

**Authors:** Marie Eve Sophie Gibson, Nathalie Fleming, Caroline Zuijdwijk, Tania Dumont

**Affiliations:** 1 University of Ottawa, Children’s Hospital of Eastern Ontario, Division of Gynecology, Ottawa, Canada; 2 University of Ottawa, Children’s Hospital of Eastern Ontario, Division of Endocrinology and Metabolism, Ottawa, Canada

**Keywords:** Adolescent, diagnosis, functional, hypothalamic amenorrhea, treatment

## Abstract

Functional hypothalamic amenorrhea (FHA) is a common cause of amenorrhea in adolescent girls. It is often seen in the setting of stress, weight loss, or excessive exercise. FHA is a diagnosis of exclusion. Patients with primary or secondary amenorrhea should be evaluated for other causes of amenorrhea before a diagnosis of FHA can be made. The evaluation typically consists of a thorough history and physical examination as well as endocrinological and radiological investigations. FHA, if prolonged, can have significant impacts on metabolic, bone, cardiovascular, mental, and reproductive health. Management often involves a multidisciplinary approach, with a focus on lifestyle modification. Depending on the severity, pharmacologic therapy may also be considered. The aim of this paper is to present a review on the pathophysiology, clinical findings, diagnosis, and management approaches of FHA in adolescent girls.

## Introduction

Functional hypothalamic amenorrhea (FHA) is defined as the absence of menses, caused by a suppression of the hypothalamic-pituitary-ovarian (HPO) axis, in which no anatomic or organic cause is found (1). It is potentially reversible, and is often seen in the setting of stress, weight loss, or excessive exercise (1,2,3). FHA can present as either primary or secondary amenorrhea. Primary amenorrhea is defined as the absence of menarche by age 15 in the presence of mature breast development, or three years after thelarche (4). Delayed puberty is defined as the absence of thelarche by the age of 13 (4). Secondary amenorrhea is defined as the absence of menses for more than three cycles in someone who was previously menstruating regularly, or longer than six months in someone with irregular cycles (5,6). FHA is the most common form of primary and secondary amenorrhea in adolescent girls (7). With specific regard to secondary amenorrhea, FHA and polycystic ovarian syndrome (PCOS) are the most common causes, other than pregnancy (1). If prolonged, FHA has potential consequences for metabolic, bone, cardiovascular, mental, and reproductive health. This article will highlight what is known about the pathophysiology of FHA, as well as the necessary steps in evaluating a patient for FHA, and the important aspects of its management.

## Pathophysiology

FHA is caused by a suppression of the HPO axis. In normal puberty, gonadotropin-releasing hormone (GnRH) is released by the hypothalamus in a pulsatile fashion, and stimulates both the synthesis and secretion of luteinizing hormone (LH) and follicle stimulating hormone (FSH) from the anterior pituitary (7). In patients with FHA, studies have shown that GnRH secretion is suppressed, LH pulsatility is impaired (8,9,10,11), and total LH and FSH levels are reduced (11,12,13,14). FHA is therefore classified as a form of hypogonadotropic hypogonadism, which results in a hypoestrogenic state (8,12,13,14). In FHA, suppression of the HPO axis is caused by common triggers including psychological stress, disordered eating, weight loss, and excessive exercise (1,2,3).

Though amenorrhea is often associated with eating disorders such as anorexia nervosa, FHA is often found to be the underlying etiology for amenorrheic patients who maintain 90-110% of their ideal body weight (IBW) and who do not meet diagnostic criteria for an eating disorder (15). IBW is calculated by the Devine formula [IBW (kg)=45.5 kg + 2.3 kg for each inch over 5 feet] (16) or can be determined by standardized height and weight tables such as the Metropolitan Life tables (17). Disordered eating is quite common in adolescent girls. In a cross-sectional study of grade 10 girls, 4.1% of girls sampled met the criteria for secondary amenorrhea and 23% disclosed disordered eating. Of the girls with amenorrhea, 40% reported fasting or purging. Interestingly, body mass index (BMI) (BMI; kg/m2) was not significantly different between those who were eumenorrheic or amenorrheic (18). Studies have shown that patients with FHA exhibit more cognitive restraint (19), drive for thinness (12,19,20,21), and purging behaviours (21,22) compared to eumenorrheic controls.

Excessive exercise has been linked to the development of FHA (23,24). In one study, rates of secondary amenorrhea were three times higher in athletes compared to controls, with the highest rates seen in long distance runners (25). Since the early 1990s, the Female Athlete Triad (FAT) has been used to describe athletes who also present with disordered eating, osteoporosis, and amenorrhea (26). In 2017, the American College of Obstetricians and Gynecologists revised the definition of FAT to be more inclusive. The criteria are now: low energy availability with or without disordered eating, menstrual dysfunction, and low bone density (27). Though the menstrual dysfunction in FAT is thought to be hypothalamic in nature, FAT differs from FHA because athletes are not required to be amenorrheic to meet criteria for FAT. Moreover, not all patients with FHA are athletes or meet the criteria for FAT.

Onset of amenorrhea can also be seen in the setting of stress (12,28,29,30). In a study of adolescent girls with FHA, identified stressors included common life events such as changing schools, newly engaging in sexual activity, and breaking up with a boyfriend. Chronic illness of a family member and the death of a friend were also observed. Lastly, 50% of the adolescents in this study described family conflict (12). Patients with FHA have also been shown to cope less well with stress, including their autonomic responses, compared to those with PCOS and eumenorrheic controls (31).

Lastly, there may also be a genetic basis to the development of FHA. One study identified six heterozygous gene mutations in patients with FHA that are shared among patients who have congenital (idiopathic) hypogonadotropic hypogonadism, suggesting a possible vulnerability to the effects of stressors on the HPO axis. Mutations found involved the fibroblast growth factor receptor 1 gene *FGFR*, the prokineticin receptor 2 gene *PROKR2*, the GnRH receptor gene *GNRHR*, and the Kallmann syndrome 1 sequence gene *KAL1*. Such mutations were not found in healthy controls (32).

Regardless of the trigger for FHA, a common hypothesis is that an increase in corticotropin-releasing hormone (CRH), in response to stress, suppresses GnRH pulsatility (10). Patients with FHA have increased cortisol levels (10,12,13,14,20,29,33), as well as blunted responses to the injection of human CRH (hCRH) (13,29,33). In addition, the neurotransmitter ƴ-aminobutyric acid has also been linked to suppression of GnRH (13). Thyroid hormone changes are also noted in FHA. Patients with FHA tend to have lower total triiodothyronine (T3) and total thyroxine (T4) concentrations compared to eumenorrheic controls (11,34). However, their concentrations of free T3 and T4 may remain intact due to lower affinity of thyroid binding globulin (34). Thyroid-stimulating hormone (TSH) levels typically remain normal (11,14,34) and patients appear to be clinically euthyroid (34). Metabolic disturbances are also observed, with decreased leptin (8,12,14,19,35,36), decreased fasting insulin (12,14,35), decreased insulin-like growth factor-1 (IGF-1) (8,12), increased fasting peptide YY (19), and increased fasting ghrelin in patients with FHA (19,22). These changes reflect the overall energy deficit in patients with FHA.

## Diagnosis of FHA

The diagnosis of FHA can be challenging in adolescents, as this is commonly a time when the HPO axis is developing. However, primary amenorrhea should always be investigated, as 98% of girls will achieve menarche by the age of 15 (37). Furthermore, 90% of menstrual cycles will range between 21-45 days, even in the first few post-menarchal years (38), highlighting the importance of investigating secondary amenorrhea in this age group. As FHA is a non-organic cause of amenorrhea, it is often considered a diagnosis of exclusion. Table 1 summarizes the vast differential diagnoses of amenorrhea, which should be taken into consideration.

**History:** A pubertal history should include onset and timing of breast and pubic hair development, as well as growth spurt. A detailed menstrual history should be obtained to characterize the type of amenorrhea and its onset. One should look for possible triggers including stressful life events, disordered eating, weight loss (regardless of initial weight), or excessive exercise. Disordered eating can include avoidance of certain foods (typically foods high in fat, sugar, and calories), restricting, and/or purging (self-induced vomiting, laxative use, or compensatory exercising). A diet log can be helpful. If weight loss has been identified as a contributing factor, it is important to note the weight at which the patient became amenorrheic and the tempo of the weight loss. Lastly, it is important to inquire about how the weight loss was achieved, as well as how they feel about the weight loss, as this helps determine whether a formal eating disorder diagnosis should be considered. The type of exercise should be noted, as well as the duration and intensity. Patients should be asked about their past medical history, including chronic illness or malignancy. A list of medications should be obtained, and previous or current treatments with chemotherapy or radiation should be noted. A sexual history, taken alone with the adolescent in complete privacy, should be obtained, including use of contraceptives. On review of symptoms, patients should be asked about possible associated symptoms in a head to toe approach. To reiterate, one should ask about possible triggers affecting the hypothalamus, such as stress, disordered eating, weight loss, or excessive exercise. Headaches, visual disturbances, or galactorrhea could suggest the presence of a prolactinoma or another central nervous system disorder. A history of anosmia could point to Kallman syndrome. Changes in energy, temperature regulation, or bowel movement frequency could be related to an underlying thyroid disorder. Patients should be asked about signs of hyperandrogenism, such as acne or hirsutism, as this could point to a diagnosis of PCOS or late-onset congenital adrenal hyperplasia. More significant virilization (clitoromegaly, severe hirsutism, voice changes) could point to an androgen secreting tumour of either adrenal or ovarian origin. Vasomotor symptoms such as hot flashes or night sweats could be indicative of primary ovarian insufficiency (POI). Inquire about symptoms of pregnancy, such as weight gain, nausea, fatigue, vomiting, or breast tenderness. Abdominal pain, either cyclic or chronic, could indicate a possible Müllerian anomaly. Lastly, a thorough family history, including the menstrual history of the biological mother, should be obtained. Questions about possible triggers and sexual history should be reserved for the confidential portion of the interview. Commonly, the “Home environment, Education and employment, Eating, peer-related Activities, Drugs, Sexuality, Suicide/depression, and Safety from injury and violence-HEEADSSS” format is used (39).

**Physical examination:** The physical examination should first begin with a general inspection of the patient’s well being. The patient’s height and weight should be measured and plotted on growth curves that ideally have previously been completed by the referring or primary care provider in order to facilitate comparisons and trends. The BMI (kg/m^2^) should be calculated and plotted. Vital signs should include blood pressure and heart rate. Hypertension and tachycardia can be seen in hyperthyroidism or Cushing syndrome, whereas hypotension and bradycardia can be seen in hypothyroidism, adrenal insufficiency, and severe eating disorders. Look for stigmata of Turner syndrome (low hairline, webbed neck, wide carrying angle, shield chest, and nevi, including facial nevi). Look for signs of restrictive or purging behaviours, which include cachexia, erosion of dental enamel, parotid gland swelling, vellus hair, Russell’s sign (calluses on the knuckles) and hypercarotenemia (yellowing of the skin). A visual field examination and fundoscopy is recommended, particularly if there are concerns regarding central nervous system symptoms in the history. Palpate the thyroid gland for a goiter or nodules and examine for other signs of thyroid disease (exophthalmos or proptosis, lid lag, hair or nail changes). Palpate the abdomen for masses. Look for signs of insulin resistance (acanthosis nigricans), hyperandrogenism (acne or hirsutism), or virilization (male pattern hair loss, change in muscle mass distribution, clitoromegaly, or voice deepening). Complete Tanner staging should be done to document pubertal development (40). The papilla and surrounding breast may also be examined for residual signs of galactorrhea. Perform an external genital examination with the aid of labial traction to assess for a patent hymen and lower vagina. This examination can also aid in determining the extent of estrogenization of the vulva. Typically, a reddened and thin hymen is seen in an estrogen-deficient state, whereas a light pink and plumper hymen is seen in the presence of adequate estrogen levels. The presence of leukorrhea can also point to adequate estrogenization. Lastly, a bimanual examination can be performed in patients who are sexually active, to palpate for a uterus and to rule out an adnexal mass. Typically, patients with FHA will have a physical examination within normal limits.

**Endocrinological investigations:** Initial blood work-up should include measurement of the beta subunit of human chorionic gonadotropin concentration, regardless of the disclosed sexual history, to rule out pregnancy. FSH, LH, estradiol, prolactin, and TSH concentrations should also be measured routinely. If there are signs of hyperandrogenism on examination, an androgen panel should be ordered, including total and free testosterone, androstenedione, and dehydroepiandrosterone sulfate, along with a 17-hydroxyprogesterone concentration, preferably in the early morning (1,3,41). Assessment of cortisol status may also be considered, based on presenting features. See Table 2 for a summary of laboratory findings in FHA.

A progesterone withdrawal challenge can be given to aid in the diagnosis. Five to 10 mg of medroxyprogesterone acetate are given for five to 10 days, after which the patient should experience a withdrawal bleed (41). A positive test is indicated by vaginal bleeding within two to seven days of completing the course of progestin (6). A negative test, or a lack of bleeding, may suggest an outflow tract abnormality or a hypoestrogenic state, as estrogen is responsible for thickening the endometrial lining (43). Scant withdrawal bleeding or spotting suggests marginal levels of endogenous estrogen production (6). Unfortunately, experts caution routine use of the progesterone withdrawal challenge, as it may be unreliable in determining the degree of estrogenization as this test is associated with false negative withdrawals (1,3,43,44).

**Radiological investigations:** An ultrasound of the pelvis is helpful to identify the presence of a uterus and ovaries, and to rule out an adnexal mass. If a Müllerian anomaly is suspected, magnetic resonance imaging (MRI) of the pelvis, or a 3D transvaginal ultrasound, if the patient is coitarchal, may better characterize the specific anomaly (45,46,47). Head imaging with computed tomography or MRI is not typically required unless the adolescent girl presents with galactorrhea (+/- hyperprolactinemia), headaches or visual disturbances, suggesting a possible intracranial lesion (1,41,48). It may also be indicated if there is a negative progesterone withdrawal challenge (4).

Due to the risk of osteopenia and osteoporosis associated with hypoestrogenism, patients with prolonged amenorrhea, of six months or more, should be considered for baseline bone mineral density (BMD) assessment measured by dual-energy X-ray absorptiometry (DEXA/DXA) scan and lateral spine radiograph to assess for asymptomatic vertebral fractures (15,41,49,50,51,52,53). In adolescents, BMD Z-scores are used as these values are adjusted for age and gender. They must also be further interpreted in relation to the patient’s body size, ethnicity, and pubertal staging or skeletal maturity (defined by bone age) (53). There is no absolute BMD Z-score threshold that can be used alone to define osteoporosis. Rather, a diagnosis of osteoporosis requires the presence of both a clinically significant fracture history (≥3 long bone fractures at any age up to 19 years old) and a BMD Z-score <-2.0. However, a BMD Z-score >-2.0 does not to preclude the possibility of skeletal fragility, and in the setting of a low-trauma vertebral fracture, there is no BMD Z-score requirement to make a diagnosis of osteoporosis (54). Evaluation of the BMD Z-score trajectory, based on serial measurements over time, provides valuable information about which patients are at risk for fractures (declining BMD Z-scores), versus those who may be showing signs of recovery (53). BMD should be repeated every six to 12 months to assess for trajectory of BMD Z-score, in patients where risk factors remain present. Spine radiographs should also be monitored at a similar interval to assess for asymptomatic vertebral fracture (or immediately if symptomatic), particularly if there is decline in BMD Z-score (53).

**Other investigations:** A karyotype should be performed if a chromosomal abnormality, such as Turner syndrome is suspected and/or if gonadotropins are elevated. If gonadotropins are elevated and POI is diagnosed, other testing would be required including autoimmune antibodies and Fragile X testing.

## Management

The menstrual cycle has been recognized as an important vital sign in adolescent girls (55,56), and the absence of menses may be an indication of compromised overall health. As such, the main goal of management in FHA is the resumption of menses.

**Lifestyle modification:** Addressing possible triggers such as weight loss, disordered eating, or excessive exercise is a primary focus in the management of FHA. In one study by Kondoh et al (29), patients with FHA related to weight loss were treated by a nutritionist for at least six months. 54.0% of these patients resumed menses with an average recovery time of 19.4±5.0 months. A small yet statistically significant increase in BMI was observed before resumption of menses in these patients. However, there has been debate over whether a critical increase in BMI is required to resume menses. A common recommendation in the literature is that a 1-2 kg weight gain from current weight, or a 5% increase in body weight, can result in the resumption of menses and improve BMD in patients with FHA. This recommendation is based on two small studies (57,58). In a study by Kopp-Woodroffe et al (57), three out of four amenorrheic participants resumed menses after a 20-week program. The program involved incorporating one rest day per week and a nutritional supplement to improve overall energy balance. In another study by Lindberg et al (58), four out of seven amenorrheic participants in a 15-month program resumed menses and had a small, statistically significant increase in BMD. Their program included a reduction in exercise duration and calcium supplementation. Larger prospective studies would be beneficial in confirming these results.

Specifically in amenorrheic female athletes, a multidisciplinary approach, which includes nutritional therapy, psychological therapy, and modification of exercise regimen has been recommended (59,60).

In all patients with FHA, if lifestyle modification is the primary treatment modality, a follow up should be done every two to three months to determine whether the desired effect is being achieved (60).

**Psychological therapy:** Adolescent girls and young adult women with FHA have been shown to cope less well with stress (31), and are also at a higher risk of depression (50). In the study by Kondoh et al (29), patients with FHA related to psychogenic stress, aged 15-33, were treated with psychoeducation which focused on stress management. A greater proportion of these patients recovered compared to those with weight-associated FHA; 81.8% versus 54.0%. Their average time to recovery was also slightly shorter at 17.2±4.1 months versus 19.4±5.0 months. A small randomized controlled trial (RCT) looked at the effect of a 20-week intervention with cognitive based therapy (CBT) in patients with FHA (61). In this study, the eight patients randomized to the CBT arm had a higher rate of ovarian activity (87.5%) compared to those eight patients that were in the observation arm (25.0%). Ovarian activity was determined by measuring plasma estradiol and progesterone levels, in order to confirm ovulation. BMI did not significantly change during the intervention. CBT has also been shown to have an impact on metabolic health in these patients. In a follow-up study by Michopoulos et al (62), patients randomized to the CBT arm had an improvement in cortisol, TSH, and leptin concentrations compared to those in the observation arm.

Other forms of psychological therapy have been studied. In a small prospective study, 12 patients with FHA, aged 20-33, were given a 45-70 minute hypnotherapy session and then observed for 12 weeks (63). Nine patients (75%) resumed menses, and one patient became pregnant during this time. All patients also reported increased general well-being and improved self confidence.

Though studies looking at psychological therapy in FHA have been small, the effects of therapy are promising and are unlikely to result in harm. Therefore, psychological therapy may be considered as part of the multidisciplinary treatment of patients with FHA.

**Pharmacological therapy:** The main role of pharmacological therapy in FHA is to promote bone health and prevent the development of osteoporosis. A lack of estrogen during premenopausal years has been linked to decreased BMD. This is based on studies looking at the outcomes of premenopausal women undergoing bilateral oophorectomy (64,65). In one study, vertebral bone loss could be detected as early as six months post-operatively (64). An increase in the frequency of fragility fractures of the radius and femoral neck was also observed (65). Similarly, in patients with FHA, the associated hypoestrogenic state can result in reduced bone density (15,50,51). In young women less than 20 years of age, missing even 50% of menstrual cycles can result in a significant decrease in BMD (52). Therefore, studies have looked at the effects of hormone replacement therapy on BMD in patients with FHA.

A systematic review by Liu and Lebrun (66) summarized ten studies evaluating the impact of hormone therapy on BMD in women with FHA. They found seven studies which demonstrated a positive effect of combined oral contraceptives (COCs) on BMD (67,68,69,70,71,72,73), two studies that showed no effect (74,75), and one case report where a negative effect was observed (76). Of the studies that showed a positive effect, two were small RCTs (67,68). Hergenroeder et al (67) showed a significant increase in both the total BMD and lumbar spine BMD of five patients receiving 35 µg ethinyl estradiol (EE) + 0.5-1 mg norethindrone, compared to five controls. Castelo-Branco et al (68) showed a significant increase in lumbar spine BMD in 24 patients taking 30 µg EE + 0.15 mg desogestrel and 22 patients taking 20 µg EE + 0.15 mg desogestrel, compared to 18 control patients who showed a decrease in BMD. Of the studies that showed no effect, one cohort study looking at female long distance runners, found no difference in BMD after one year in nine patients who started on a COC (75). However, in the same study 11 patients with FHA who were not using a COC showed a significant reduction in BMD over the same time period. Currently, the Endocrine Society has recommended against using COCs for the sole purpose of improving BMD, due to conflicting evidence. Instead, a trial of short-term transdermal estrogen with a cyclic oral progestin is recommended in amenorrheic adolescents who have not been successful with lifestyle modification, and who are not in need of COCs for contraception (41).

To date, the majority of evidence for the positive effects of transdermal estrogen on BMD comes from research involving patients with anorexia nervosa (77,78). However, its use in patients with FHA is attracting interest and has started to be studied. Zanker et al (76) published a case report of a 24-year-old amenorrheic athlete, whom they followed for 12 years. They measured her body weight every three months and her BMD by DXA every 11-13 months. After being on COCs for five years, the BMD of her lumbar spine and proximal femur declined by 9.8% and 12.1%, respectively. Her weight dropped concomitantly from 45.1 to 41.4 kg. Over the next 3.7 years, she was treated with transdermal estrogen and an oral progestin. Her lumbar spine BMD gradually increased by 9.4%, despite a further 0.8 kg decline of body mass. In the last 2.9 years of the study, she continued the transdermal estrogen, gained a total of 8.1 kg of body mass, and had a 16.9% increase in her proximal femur BMD. Furthermore, an RCT by Ackerman et al. (79) from 2019 showed an improvement in BMD in athletes with oligo-amenorrhea receiving transdermal estrogen. In this study, 43 patients were randomized to receive a 100 mcg 17b-estradiol transdermal patch twice weekly with cyclic micronized progesterone (200 mg, 12 days per month), 40 patients to receive a daily pill with 30 µg EE + 0.15 mg desogestrel, and 38 patients received no hormonal treatment. All patients also received 800 IU of vitamin D and ≥1200 mg of calcium per day. BMD was assessed at baseline, six, and 12 months. Patients randomized to the patch arm had significantly higher spine and femoral neck BMD Z-scores at 12 months compared to the pill and the no treatment arm, and higher hip BMD Z-scores than the pill arm. The results of this landmark study are promising and lend support to the use of transdermal estrogen in patients with FHA.

In amenorrheic adolescents, 1200-1500 mg of calcium supplementation (80) as well as vitamin D 400-1000 IU (1) are recommended daily to support bone health. However, other therapies such as testosterone or bisphosphonates are not currently recommended to improve BMD in patients with FHA (41,81), as the literature available focuses mainly on patients with anorexia nervosa and the current evidence is limited.

**Fertility:** Patients with FHA may experience escape ovulation and therefore contraception is important if they do not desire pregnancy (41). In addition, adolescents with FHA may inquire about future fertility. Ovarian reserve is typically normal in these patients, as evidenced by their normal anti-Müllerian hormone (AMH) levels (42). In patients who desire pregnancy, ovulation induction with pulsatile GnRH is the current gold standard (82,83,84,85). When compared to injectable gonadotropins, chances of conception are higher after six cycles of pulsatile GnRH at 96% versus 72% for injected gonadotropins based on life table analysis (82). Furthermore, injectable gonadotropins are associated with a higher rate of multiples (14.8% versus 9.3%), though the finding was not statistically significant (82). These results were more recently replicated in a study by Dumont et al (84) which showed a per patient conception rate of 65.8% with pulsatile GnRH versus 23.5% with gonadotropins. Though the trend favouring pulsatile GnRH is the same in both studies, the conception rates in the Dumont et al study are significantly lower. This may be explained by the differences in study populations between these studies, with lower BMI and baseline gonadotropin levels in the Dumont et al (84) study. The mean BMI in Dumont et al was 18.5 kg/m^2^ (pulsatile GnRH group) and 18 kg/m^2 ^(gonadotropin group), whereas in Martin et al (82) it was 24.3 kg/m^2^ (pulsatile GnRH group) and 24.5 kg/m^2^ (gonadotropin group). Baseline LH, FSH, and estradiol levels were also lower in Dumont et al (84). Naltrexone, an opioid antagonist, has also been studied. GnRH secretion has been found to be suppressed by endogenous opioids (86). It was hypothesized that GnRH pulsatility could therefore be stimulated by opioid antagonism. Though naltrexone has been shown to increase GnRH pulsatility and increase rates of ovulation (86,87,88,89), its use has not become standard practice.

**Cardiovascular considerations:** Patients with prolonged FHA may be at higher risk of cardiovascular complications in the future (90). Studies in pre-menopausal adult women have shown hypothalamic hypoestrogenism is associated with a higher risk of coronary artery disease (91). Other possible effects include vascular endothelial dysfunction and reduced regional blood flow, as was shown in young amenorrheic athletes (92). These athletes were also found to have abnormal lipid profiles, including elevated total cholesterol and low-density lipoprotein cholesterol (92). As a follow-up study, Rickenlund et al (93) investigated the effects of using a COC (30 µg EE + 0.15 mg levonorgestrel) on these cardiovascular endpoints in amenorrheic athletes. While an improvement in vascular endothelial function after nine months of COC use was found, the lipid profile did not significantly change, with the exception of a small increase in high-density lipoprotein cholesterol. As this study was small, the authors indicated the need for larger, long-term studies to determine the clinical importance of their findings. As of now, the majority of recommendations surrounding cardiovascular health of patients with FHA focus on the lifestyle modifications that can be made to resume menses (90).

**Novel therapies:** Studies are now focusing on the underlying metabolic abnormalities within FHA to direct therapy. Small RCTs have looked at the effects of treatment with recombinant human leptin. Welt et al (94) demonstrated an improvement in serum estradiol, increased levels of free T4, and IGF-1 with administration of recombinant methionyl human leptin (r-metHuLeptin; starting dose 0.08 mg per kilogram of body weight per day) subcutaneously for two to three months. Three out of eight women (37.5%) resumed ovulatory cycles, which the authors stated was higher than the expected rate of spontaneous ovulation of 10%. In a small RCT, recombinant human leptin (metreleptin; starting dose 0.08 mg per kg of body weight per day) administered subcutaneously over 36 weeks, increased estradiol levels and decreased cortisol levels compared to placebo (95). Patients receiving recombinant human leptin in this study were also more likely to resume menses compared to controls (70% versus 22.2%). In both studies, markers of bone formation were also found to be increased, though BMD did not change significantly (94,95). The administration of kisspeptin has also been studied, and while acute administration appears to stimulate release of LH and FSH, chronic administration results in tachyphylaxis. Thus, the authors concluded that acute administration of kisspeptin may have therapeutic potential in patients with FHA (96). The Endocrine Society has recommended against the use of leptin or kisspeptin in the management of patients with FHA, as more research is needed in this area (41).

## Conclusion

FHA is a common cause of both primary and secondary amenorrhea in adolescent girls. Common triggers include stress, weight loss, and excessive exercise. As FHA is a diagnosis of exclusion, a comprehensive workup should be performed to rule out anatomic and organic causes of amenorrhea. Prolonged FHA can have negative consequences on many aspects of a young women’s health, including metabolic, bone, cardiovascular, mental, and reproductive implications. The main goal in these patients is the resumption of menses. Lifestyle modifications are the first line focus for adolescent girls with FHA and a multidisciplinary approach, including a pediatric gynecologist and/or endocrinologist, pediatric sport psychologist, and sport dietician is beneficial. Pharmacological therapy can be considered in order to promote bone health, with transdermal estrogen being a promising option for patients. Further research on novel agents, such as recombinant human leptin and kisspeptin, is required before considering their routine use in patients with FHA.

## Figures and Tables

**Table 1:**
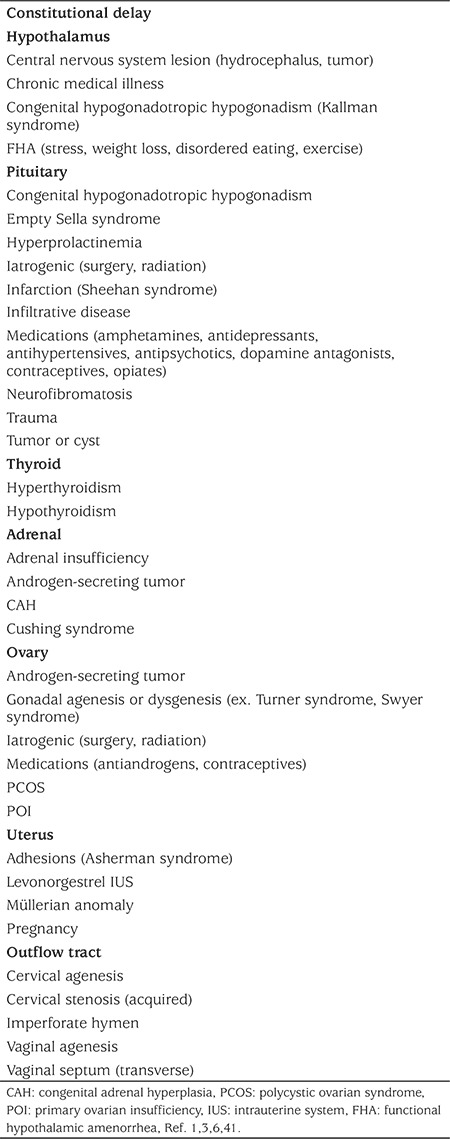
Differential diagnosis of amenorrhea

**Table 2:**
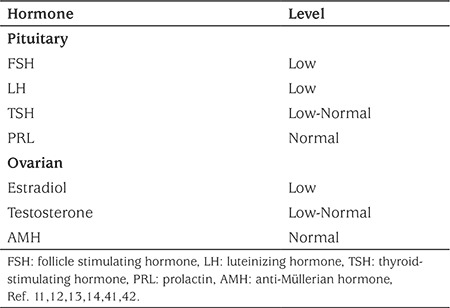
Typical hormone pattern in functional hypothalamic amenorrhea
